# CRISPR/Cas12a-Based Ultrasensitive and Rapid Detection of *JAK2* V617F Somatic Mutation in Myeloproliferative Neoplasms

**DOI:** 10.3390/bios11080247

**Published:** 2021-07-24

**Authors:** Miaomiao Chen, Chunhua Zhang, Zhiqing Hu, Zhuo Li, Menglin Li, Lingqian Wu, Miaojin Zhou, Desheng Liang

**Affiliations:** Center for Medical Genetics & Hunan Key Laboratory of Medical Genetics, School of Life Sciences, Central South University, Changsha 410078, China; chenmiaomiao@sklmg.edu.cn (M.C.); zhangchunhua@sklmg.edu.cn (C.Z.); huzhiqing@sklmg.edu.cn (Z.H.); lizhuo@sklmg.edu.cn (Z.L.); limenglin@sklmg.edu.cn (M.L.); wulingqian@sklmg.edu.cn (L.W.)

**Keywords:** *JAK2* V617F, CRISPR/Cas12a, mutation detection, fluorescence detection system, lateral flow strip, Philadelphia-negative myeloproliferative neoplasms

## Abstract

The *JAK2* V617F mutation is a major diagnostic, therapeutic, and monitoring molecular target of Philadelphia-negative myeloproliferative neoplasms (MPNs). To date, numerous methods of detecting the *JAK2* V617F mutation have been reported, but there is no gold-standard diagnostic method for clinical applications. Here, we developed and validated an efficient Clustered Regularly Interspaced Short Palindromic Repeats (CRISPR)/CRISPR associated protein 12a (Cas12a)-based assay to detect the *JAK2* V617F mutation. Our results showed that the sensitivity of the *JAK2* V617F/Cas12a fluorescence detection system was as high as 0.01%, and the *JAK2* V617F/Cas12a lateral flow strip assay could unambiguously detect as low as 0.5% of the *JAK2* V617F mutation, which was much higher than the sensitivity required for clinical application. The minimum detectable concentration of genomic DNA achieved was 0.01 ng/μL (~5 aM, ~3 copies/μL). In addition, the whole process only took about 1.5 h, and the cost of an individual test was much lower than that of the current assays. Thus, our methods can be applied to detect the *JAK2* V617F mutation, and they are highly sensitive, rapid, cost-effective, and convenient.

## 1. Introduction

Philadelphia-negative myeloproliferative neoplasms (MPNs), including polycythemia vera (PV), essential thrombocythemia (ET), and primary myelofibrosis (PMF), are a group of heterogeneous chronic diseases characterized by the clonal expansion of one or more myeloid lineages [1,2]. It was reported that the crude annual incidence rate of classic MPNs ranged from 1.15 to 4.99 per 100,000, with a prevalence rate of 93.43 per 100,000 [3]. The *JAK2* V617F mutation is the most common molecular event in the classic MPNs and presents in more than 95% of patients with PV and 50–60% of patients with ET or PMF [4,5,6,7]. In 2016, the World Health Organization (WHO) classification of myeloproliferative neoplasms specifically recognized the *JAK2* V617F mutation as one of the main diagnostic criteria of Philadelphia-negative MPNs [8].

JAK2, a non-receptor-type tyrosine kinase belonging to the Janus kinase family, is encoded by the *JAK2* gene located on chromosome 9p24, which plays an important role in the signal transduction of cytokines and several hematopoietic growth factor receptors [9,10]. Structurally, JAK2 is characterized by the presence of seven homologous kinase domains (JH1–JH7), among which the JH1 domain is the catalytic active region of JAK2 and has tyrosine kinase activity. In contrast, the JH2 domain has no kinase activity, and it negatively regulates the kinase activity of JH1 domain [11]. The *JAK2* V617F mutation is an acquired G-to-T transversion at nucleotide 1849 of exon 14, resulting in amino-acid substitution of valine (V) to phenylalanine (F) at codon 617. The mutation lies in the JH2 pseudo-kinase domain and interferes with JH2-mediated autoinhibition, leading to activation of the JAK/STAT signaling pathway. This results in abnormal cell proliferation and differentiation, eventually leading to MPNs [11,12].

As key molecular evidence for diagnosis of MPNs and a target for therapeutic intervention, the *JAK2* V617F mutation has attracted great attention. Several techniques have been utilized to detect and quantify the *JAK2* V617F mutant allele, such as allele-specific PCR (AS-PCR), DNA sequencing, PCR restriction fragment length polymorphism (PCR-RFLP), denaturing high-performance liquid chromatography (DHPLC), quantitative PCR (qPCR), and droplet digital PCR (dd-PCR) [4,13,14,15,16,17,18,19,20,21]. However, the most appropriate assay for clinical laboratories has not yet been defined [22,23]. Currently available techniques have their own pros and cons regarding the application scope, detection performance, and testing cost [24]. However, most of these techniques are inconvenient for popular, promotion, and detection use in the field, since they are time-consuming and labor-intensive, and they may require costly or specialized equipment and highly trained personnel [25]. Therefore, there is a great demand for a rapid, easy-to-perform, sensitive, and economic detection system targeting *JAK2* V617F mutation.

Clustered Regularly Interspaced Short Palindromic Repeats (CRISPR)/CRISPR associated proteins (Cas) systems are RNA-guided adaptive immunity systems in bacteria and archaea [26,27,28], which can accurately recognize a specific sequence; they are used to detect various targets, especially pathogens [29,30,31,32,33,34]. CRISPR/Cas-based nucleic-acid detection technologies have an extremely high sensitivity, specificity, and reliability, and they can be used for point-of-care (POC) diagnostics by binding isothermal amplification and the lateral flow strip [30,31,34,35,36,37,38]. Cas12a is an important member of the Cas family for nucleic-acid detection. Cas12a is an RNA-guided Class II type V CRISPR nuclease, which cleaves target double-stranded DNA (dsDNA) following recognition of a matching dsDNA sequence containing a T-rich protospacer-adjacent motif (PAM) [39]. After cleaving the target dsDNA, Cas12a will procced to cleave surrounding single-stranded DNA (ssDNA) in a nonspecific manner, so-called “trans cleavage” activity [31,35,40]. Based on this property, Chen et al. developed the Cas12a-based nucleic-acid detection technology termed DETECTR [31]. Gootenberg et al. combined Cas13, Cas12a, and Csm6 to create the SHERLOCKv2 nucleic-acid detection platform [35]. Cas12a is not sensitive enough to directly detect low levels of nucleic acids, and the upstream amplification step can significantly improve the sensitivity of the detection [41,42]. Therefore, this study integrated the CRISPR/Cas12a system, DNA amplification (recombinase polymerase amplification or polymerase chain reaction), and a lateral flow strip (or a fluorescence detection instrument) to establish methods with excellent detection performance, low cost, and convenience, for the detection of *JAK2* V617F mutation.

## 2. Materials and Methods

### 2.1. Reagents and Instruments

The following reagents and instruments were used: primers (Sangon Biotech, Shanghai, China), CRISPR RNAs (crRNAs) (Sangon Biotech, Shanghai, China), probes (Sangon Biotech, Shanghai, China), pGEM-T Easy Vector (Promega, Madison, WI, USA), *E. coli* DH5α (ThermoFisher Scientific, Waltham, MA, USA), TwistAmp Liquid Basic Kit (TwistDx, Cambridge, UK), Cycle Pure Kit (Omega Bio-tek, Norcross, GA, USA), Premix Ex Taq HS (TaKaRa, Dalian, China), Lba Cas12a (NEW ENGLAND BioLabs, NEB, Ipswich, MA, USA), NEBuffer 2.1 (NEB, Ipswich, MA, USA), Later Flow Dipsticks (Milenia Biotec GmbH, Gießen Germany), ChamQ Universal SYBR qPCR Master Mix (Vazyme, Nanjing, China), NanoDrop 1000 spectrophotometer (ThermoFisher Scientific, Waltham, MA, USA), ProFlex PCR System (ThermoFisher Scientific, Waltham, MA, USA), and CFX-96 Quantitative Fluorescence Instrument (BioRad, Hercules, CA, USA).

### 2.2. Source of Clinical Samples and Ethics Statement

The study sample comprised a patient with essential thrombocythemia (ET) diagnosed according to the 2016 WHO criteria and 13 healthy volunteers. Peripheral blood samples of the patient and 13 healthy volunteers were obtained from The First Affiliated Hospital of Jinan University and Hunan Jiahui Genetics Hospital, respectively. Their genomic DNA (gDNA) was extracted from peripheral blood using the conventional phenol–chloroform method [43]. All study participants signed informed consent and the study was approved by the Ethics Committee of School of Life Sciences, Central South University.

### 2.3. Plasmids

The wild-type fragment containing exon 14 of the *JAK2* gene (NM_004972) was amplified from normal human gDNA by PCR using primers F and R (Table S1). The *JAK2* V617F mutation was introduced by overlap extension PCR with mutation-specific primers F, R, Fm, and Rm (Table S1). The wild-type and mutant amplified fragments were cloned into the pGEM-T Easy Vector; then, the plasmids were transformed into *E. coli* DH5α for amplification, before being extracted and verified by Sanger sequencing. All the inserted sequences in the resulting recombinant plasmids were shown in Figure S1.

### 2.4. Cell Lines

The HEL human erythroleukemia cell line harboring homozygous for *JAK2* V617F mutation was purchased from the Cell Bank of the Chinese Academy of Sciences (Shanghai, China). The HEL cell line was cultured in RPMI-1640 medium supplemented with 10% fetal bovine serum (FBS) at 37 °C in a humidified atmosphere containing 95% air and 5% CO_2_. The human induced pluripotent stem (hiPS) cell line without *JAK2* V617F mutation originated from our research group and was maintained in hiPSCs medium at 37 °C under 5% CO_2_. Then, many cells were harvested, and the gDNA was isolated from HEL cells and hiPSCs using the conventional phenol–chloroform method. We further authenticated the *JAK2* V617F mutational status of two different cell lines by DNA sequencing analysis. In addition, the concentrations of DNA were measured with a NanoDrop 1000 spectrophotometer and adjusted to 100 ng/μL for storage at –20 °C until use.

### 2.5. PCR Primers and Reactions

To amplify exon 14 of the *JAK2* gene from plasmids and gDNA, PCR primers (PCR-F, PCR-R) were designed using an NCBI PrimerBlast according to the *JAK2* reference sequence (NM_004972). The primer sequences were provided in Table S1. The PCR reaction was performed in 20 μL total volume, including 16 μL of Premix Ex Taq HS, 100 nM of forward and reverse primer, 1 μL of DNA template, and nuclease-free water to final reaction volume. When using plasmids as a template for PCR amplification, the PCR cycling parameters were as follows: pre-degeneration at 95 °C for 5 min, followed by 35 cycles of 95 °C for 30 s, 58 °C for 30 s, 72 °C for 30 s, and a final extension at 72 °C for 5 min. PCR amplification of gDNA was carried out using the same oligonucleotide primers and reaction conditions, except for reducing the number of cycles to 30.

### 2.6. Recombinase Polymerase Amplification (RPA) Primers and Reactions

According to the principle of designing RPA primers and the sequence of the target gene, we used the online software (NCBI PrimerBlast) to design five forward primers and four reverse primers (Figure S2 and Table S1). A DNA sample from a healthy individual was amplified with 20 pairwise combinations of the RPA primers. All 20 amplicons were purified using the Cycle Pure Kit and screened by 1.2% agarose gel electrophoresis. We found that the RPA-F2/RPA-R3 combination was the best performing primer pair. In order to further confirm the reliability of amplification fragments, Sanger sequencing was applied to analyze the purified RPA products.

All RPA reactions were conducted by using the Twist-Dx RPA Kit according to the manufacturer’s instructions. The amplification reactions were set up in a 50 μL volume, comprising 25 μL of 2× reaction buffer, 9.2 μL of dNTPs (10 μM), 5 μL of basic E-mix, 2.4 μL of each RPA primer (10 μM), 2.5 μL of 20× core reaction mix, 2.5 μL of MgOAc (280 mM), and 1 μL of target dsDNA; then, they were incubated at 40 °C for 40 min.

### 2.7. AS-PCR Primers and Reactions

For the detection of *JAK2* V617F by AS-PCR, AS-PCR primers (Table S1) were synthesized by following previous reports [4]. The volume of the AS-PCR reaction was 20 μL, containing 10 μL of ChamQ Universal SYBR qPCR Master Mix, 5 μL of nuclease-free water, 2 μL of reverse AS-PCR primer (10 μM), 1 μL of two forward primers (10 μM), and 1 μL of gDNA (100 ng/μL). Thermocycling conditions were 5 min at 95 °C, and then 30 cycles at 95 °C for 30 s, 58 °C for 30 s, 72 °C for 30 s, with elongation at 72 °C for 5 min. After amplification, we inspected the number of amplicon bands on 2.0% agarose gel electrophoresis. The primer AS-Fm was specific for the gene with *JAK2* V617F mutation; hence, the AS-PCR amplification products for DNA samples with *JAK2* V617F mutation were 203 bp and 364 bp, whereas the samples were considered to be negative for the *JAK2* V617F point mutation if only a single 364 bp band was present.

### 2.8. crRNAs

**Figure 1:**
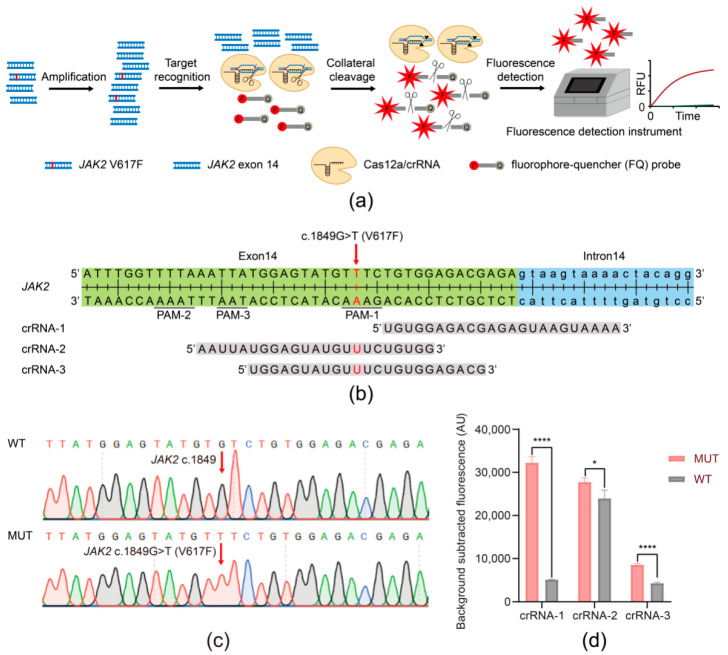
The *JAK2* V617F/Cas12a fluorescence detection system. (**a**) Schematic illustration of the *JAK2* V617F/Cas12a fluorescence detection system. The CRISPR RNA (crRNA) targeted the exon 14 of the *JAK2* gene carrying *JAK2* V617F mutation. F, fluorophore; Q, quencher. (**b**) Schematic representation of the position and sequence targeted by each crRNA: crRNA-1, crRNA-2, and crRNA-3. (**c**) Sequencing results of the mutant plasmid and wild-type plasmid for *JAK2*. MUT, mutant plasmid; WT, wild-type plasmid. (**d**) Comparison of three crRNA candidates (crRNA-1 to crRNA-3). Both the mutant plasmid concentration and the wild-type plasmid concentration were 0.01 pg/μL. MUT, mutant plasmid; WT, wild-type plasmid. Data represent means ± SD, with *n* = 3 replicates (**** *p* < 0.0001, * *p* < 0.05).

We manually designed three crRNAs (Figure 1b and Table S1) to identify the *JAK2* V617F mutation against the DNA sequences in the near proximity of the *JAK2* V617F mutation site, as well as the presence of the appropriate protospacer adjacent motif (PAM) for Cas12a (5′–TTTN and 5′–TTN) [39], and the specificity of these crRNAs was checked with the web tool CHOPCHOP [44].

### 2.9. The JAK2 V617F/Cas12a Fluorescence Assays

Each *JAK2* V617F/Cas12a fluorescence assay contained 2 μL of NEBuffer 2.1, 100 nM of Lba Cas12a, 50 nM of crRNA, 500 nM of fluorophore-quencher (FQ) probe (Table S1), 2 μL of PCR products, and nuclease-free water to a 20 μL total volume. Then, the reaction solution was mixed thoroughly and incubated at 37 °C for 1 h in the fluorescence detection instrument. The fluorescence channel was set to FAM, and the fluorescence signal was recorded every minute.

Fluorescence signals were analyzed using GraphPad Prism 8 (GraphPad Software). The date of two groups were compared using a Student’s *t*-test, and multigroup data were tested using one-way analysis of variance (ANOVA). For all analyses, statistical significance was defined as *p* < 0.05.

### 2.10. The JAK2 V617F/Cas12a Lateral Flow Strip Assays

Compared with the fluorescence detection, the lateral flow strip assays were performed using FITC–ssDNA–Biotin probes (Table S1) with commercially available lateral flow dipsticks. To explore the optimal amount of the FITC–ssDNA–Biotin probe, concentrations of the FITC–ssDNA–Biotin probes were set at 1000 nM, 500 nM, 100 nM, 10 nM, and 1 nM, and they were separately added to the 100 μL Dipstick assay buffer. After blending, the reactions and readouts on lateral flow strips were run. Accordingly, the suitable concentration of the FITC–ssDNA–Biotin probes was determined to be 100 nM.

Under the optimized amount of FITC–ssDNA–Biotin probe, The *JAK2* V617F/Cas12a lateral flow strip assay was carried out in a volume of 20 μL, including 2 μL of NEBuffer 2.1, 2500 nM of Lba Cas12a, 250 nM of crRNA, 100 nM of the FITC–ssDNA–Biotin probes, 2 μL of RPA products, and nuclease-free water to final volume. The reaction was incubated at 37 °C for 20 min. Then, 100 μL of Dipstick assay buffer was added and mixed. Subsequently, a lateral flow strip was put into the mixture vertically at room temperature. After approximately 2 min, the lateral flow strip was removed for inspection, and band intensity was directly read with naked eyes and recorded using a smartphone camera or a scanner.

## 3. Results

### 3.1. Establishing the JAK2 V617F/Cas12a Fluorescence Detection System

As shown in Figure 1a, the scheme of the *JAK2* V617F/Cas12a fluorescence detection system involved the integration of PCR amplification with Cas12a-mediated cleavage. In more detail, the specific crRNA guided the Cas12a endonuclease to bind and cleave the amplified target dsDNA. Afterward, the nonspecific ssDNA trans cleavage activity of Cas12a was activated, which cut the ssDNA-FQ probes, leading to the generation of strong fluorescence signals. Then, statistical analyses of fluorescence dates were used to determine whether the target DNA was in the DNA sample.

Cas12a activity has been shown to be strongly affected by the sequence of the PAM and crRNA [31,45,46]. Therefore, we manually designed three different crRNAs (Figure 1b and Table S1), crRNA-1, crRNA-2, and crRNA-3. The three Cas12a/crRNA complexes all recognized the sequences with *JAK2* V617F mutation. Afterward, we constructed two plasmids containing *JAK2* V617F mutation (MUT) and wild-type (WT) *JAK2*. The successful construction of the two plasmids was confirmed through Sanger sequencing (Figure 1c). We tested the three crRNAs on PCR products of the recombinant plasmids carrying exon 14 of the *JAK2* gene. The results indicated that all crRNAs were able to distinguish between mutant and wild-type *JAK2*, while the group with crRNA-1 revealed a higher specificity than the others (Figure 1d). As a result, crRNA-1 was adopted for the rest of the study.

### 3.2. The Viability and Sensitivity Evaluation of the JAK2 V617F/Cas12a Fluorescence Detection System Using Recombinant Plasmid

**Figure 2:**
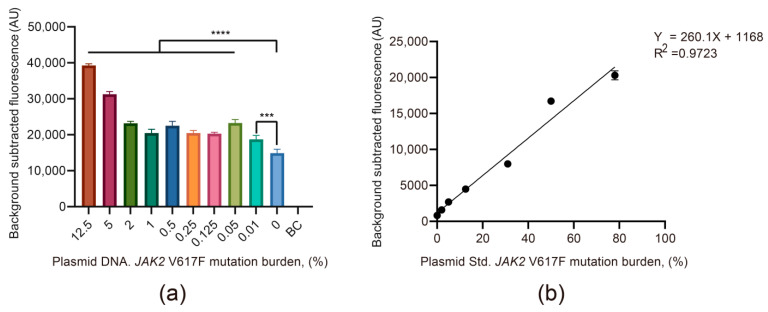
The viability and sensitivity evaluation of the *JAK2* V617F/Cas12a fluorescence detection system using recombinant plasmid. (**a**) Fluorescence signals of mixed plasmid DNA with different mutation ratios. The concentration of plasmid DNA in each sample was 0.01 pg/μL. BC, blank control. Data represent means ± SD, with *n* = 3 replicates (**** *p* < 0.0001, *** *p* < 0.001). (**b**) The linear relationship between the mutation ratio of mixed plasmid DNA and the fluorescence signal. The concentration of plasmid DNA in each sample was 0.01 pg/μL. The mutation ratio range in which the calibration cure was linear was 0–78% (*R*^2^ = 0.9723). 0%, wild-type plasmid DNA.

To determine the assay sensitivity, the successfully constructed recombinant plasmids were diluted to 0.01 pg/μL using nuclease-free water (the copies of 0.01 pg of recombinant plasmid DNA are comparable to the copies of 100 ng of human gDNA), and 0.01 pg/μL mutant and wild-type plasmid DNA was mixed in 10 different ratios (proportion of the mutant plasmids: 12.5%, 5%, 2%, 1%, 0.5%, 0.25%, 0.125%, 0.05%, 0.01%, and 0%). Subsequently, the 10 mixed plasmids and blank control group (nuclease-free water) were amplified by PCR with primers PCR-F and PCR-R (Table S1), and the PCR products were detected using the *JAK2* V617F/Cas12a fluorescence detection system. Measurement with the fluorescence detection instrument showed that the mixed plasmids containing 0.01% mutant plasmid DNA could be effectively detected by the *JAK2* V617F/Cas12a fluorescence detection system when the concentration of plasmid was 0.01 pg/μL (Figure 2a).

Then, we further investigated the potential of quantitative analysis using the *JAK2* V617F/Cas12a fluorescence detection system. According to six scaled standards of *JAK2* V617F mutant allele (2%, 5%, 12.5%, 31%, 50%, and 78%), the recombinant plasmid pair was mixed at various proportions to obtain the six diluents to create standard curves, with correlation coefficients of 0.9723 (Figure 2b). This demonstrated that the *JAK2* V617F/Cas12a fluorescence detection system had great potential in the quantitative analysis of the *JAK2* V617F allele burden.

### 3.3. The Viability and Sensitivity Evaluation of the JAK2 V617F/Cas12a Fluorescence Detection System Using gDNA Extracted from Cells

**Figure 3:**
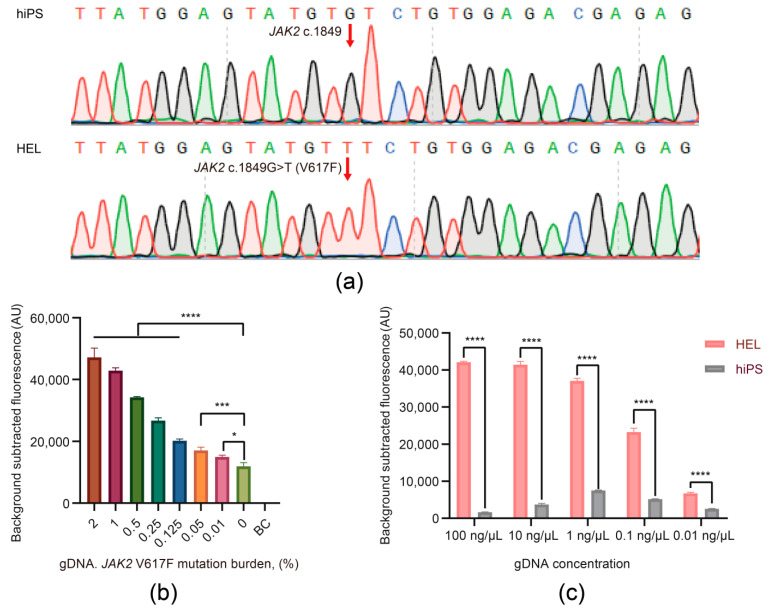
The viability and sensitivity evaluation of the *JAK2* V617F/Cas12a fluorescence detection system using genomic DNA (gDNA) extracted from cells. (**a**) Sequencing results of the HEL cell line gDNA and human induced pluripotent stem (hiPS) cell line gDNA for *JAK2*. hiPS, hiPS cell line gDNA; HEL, HEL cell line gDNA. (**b**) Fluorescence signals of mixed gDNA with different mutation ratios. The concentration of gDNA in each sample was 100 ng/μL. BC, blank control. Data represent means ± SD, with *n* = 3 replicates (**** *p* < 0.0001, *** *p* < 0.001, * *p* < 0.05). (**c**) The limit of detection of the *JAK2* V617F/Cas12a fluorescence detection system. hiPS, hiPS cell line gDNA; HEL, HEL cell line gDNA. Data represent means ± SD, with *n* = 3 replicates (**** *p* < 0.0001).

In order to better mimic the *JAK2* V617F mutation status within human gDNA, we directly exploited the gDNA derived from cells as the input and further studied the utility of the *JAK2* V617F/Cas12a fluorescence detection system for detection of the *JAK2* V617F mutation. We selected the HEL cell line homozygous for *JAK2* V617F mutation as the positive standard and the hiPS cell line homozygous for wild-type *JAK2* as the negative control, whereby the *JAK2* V617F mutation status of gDNA derived from both cell lines was confirmed by Sanger sequencing (Figure 3a). The sensitivity of the system was assessed using serial dilutions of the gDNA from the HEL cell line mixed to gDNA from hiPS cell line (the ratio of the HEL cell line gDNA in mixed gDNA: 2%, 1%, 0.5%, 0.25%, 0.125%, 0.05%, 0.01%, and 0%), where the concentration of gDNA extracted from two cell lines was 100 ng/μL. The results suggested that the system was able to reliably detect 100 ng/μL of mixed gDNA with 0.01% *JAK2* V617F allele burden (Figure 3b), and it was largely consistent with the results of the recombinant plasmids mentioned above.

Furthermore, the gDNAs from the HEL and hiPS cell lines were diluted from 100 ng/μL to 0.01 ng/μL by 10-fold gradient dilution. The *JAK2* V617F/Cas12a fluorescence detection system combined with PCR pre-amplification could detect as low as 0.01 ng/μL (~5 aM, ~3 copies/μL) of target gDNA according to the concentration gradient test results (Figure 3c). This suggested that the limit of detection (LOD) in gDNA could reach ~3 copies/μL, showing an ultrahigh sensitivity close to single-copy level.

### 3.4. Establishing the JAK2 V617F/Cas12a Lateral Flow Strip Assay

**Figure 4:**
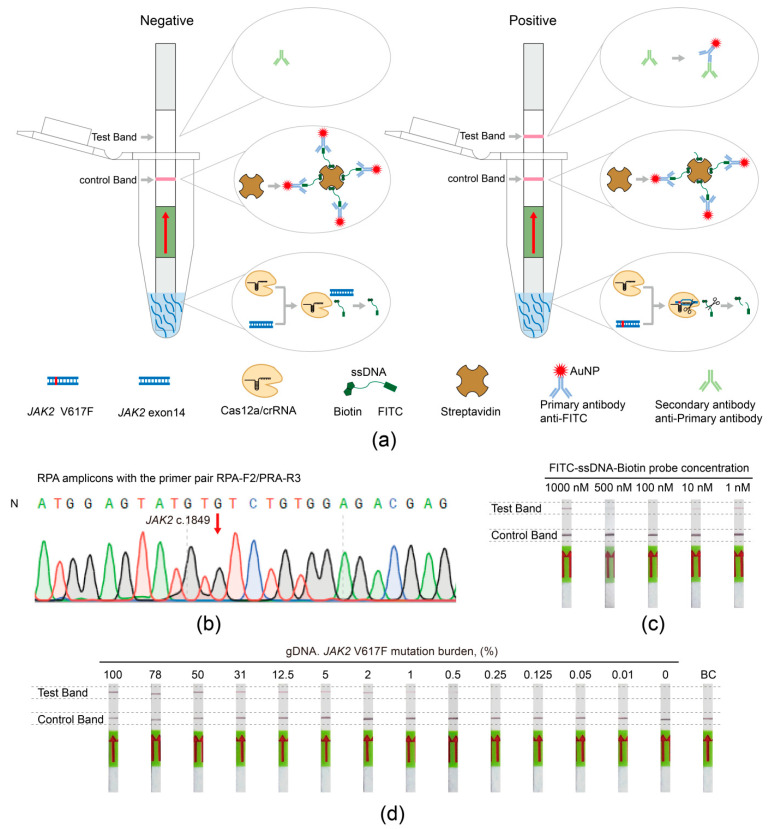
The *JAK2* V617F/Cas12a lateral flow strip assay. (**a**) Schematic illustration of the *JAK2* V617F/Cas12a lateral flow strip assay. (**b**) Sequencing results of Recombinase Polymerase Amplification (RPA) products amplified with the primer pair RPA-F2/RPA-R3. N, healthy donor. (**c**) Optimizing the amount of FITC–ssDNA–Biotin probe. (**d**) Sensitivity of the *JAK2* V617F/Cas12a lateral flow strip assay. The concentration of gDNA in each sample was 100 ng/μL. An equal volume of nuclease-free water was used as blank control.

To more simply and rapidly detect *JAK2* V617F mutation from the clinical samples in the field, we combined RPA with lateral flow strip detection to realize instrument-free visualization. The working principle of the *JAK2* V617F/Cas12a lateral flow strip assay is illustrated in Figure 4a. All lateral flow strips contained a control band with biotin ligand and a test band with anti-rabbit antibody, in addition to carrying gold particle-labeled anti-FITC antibodies to show the readout. When the amplified DNA did not contain *JAK2* V617F mutation, without trans cleavage, all FITC–ssDNA–Biotin probes remained intact and were captured by the biotin ligands at the control band, and then the FITCs were recognized and bound by all gold particle-labeled anti-FITC antibodies. Therefore, the control band generated a color signal, but the test band did not. When the amplified DNA contained *JAK2* V617F mutation, upon recognition of the matching target, the Cas12a/crRNA-1 complex cleaved the ssDNA probes. The cleaved ssDNA with the gold particle-labeled anti-FITC antibodies flowed to the test band and were captured by the anti-rabbit antibodies, followed by the formation of a color deposit on the test band. In short, the *JAK2* V617F/Cas12a lateral flow strip assay showed a negative result with a pink-colored line only at the control band and a positive result with coloration of both control and test bands.

Before establishing the assay, we optimized the RPA primers and the concentration of the FITC–ssDNA–Biotin probes. Primer pair RPA-F2/RPA-R3 was screened from 20 primer pairs, and the RPA product amplified with the primer pair RPA-F2/RPA-R3 was confirmed by Sanger sequencing (Figure 4b and Figure S2). For optimizing the concentrations of the FITC–ssDNA–Biotin probes, we set several different concentrations from 1 nM to 1000 nM and found that only the group with the FITC–ssDNA–Biotin probe concentration of 100 nM did not produce a false-positive signal (Figure 4c). Therefore, we chose primer pair RPA-F2/RPA-R3 as the primer for RPA amplification and 100 nM as the concentration of the FITC–ssDNA–Biotin probes to establish the *JAK2* V617F/Cas12a lateral flow strip assay.

### 3.5. The Viability and Sensitivity Evaluation of the JAK2 V617F/Cas12a Lateral Flow Strip Assay Using gDNA Extracted from Cells

Similarly, we analyzed the viability and sensitivity of the *JAK2* V617F/Cas12a lateral flow strip assay using mixed gDNA with different the *JAK2* V617F allele burden. The assay was proven to be able to detect the mutation of *JAK2* V617F at a low ratio of 0.5% (Figure 4d), which far exceeded the sensitivity of 1–3% required in the clinic [23]. An ultrahigh sensitivity is essential for the early diagnosis and monitoring of MPNs with *JAK2* V617F mutation.

### 3.6. The Application of the JAK2 V617F/Cas12a Lateral Flow Strip Assay in Clinical Samples

**Figure 5:**
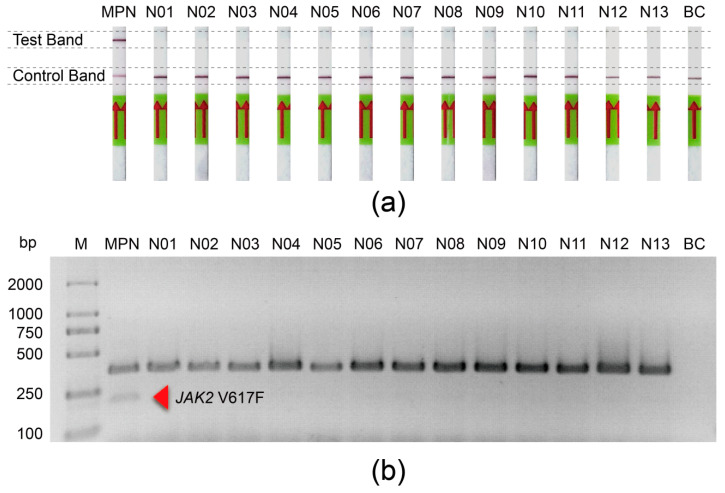
The application of the *JAK2* V617F/Cas12a lateral flow strip assay in clinical samples. (**a**) Response signals of the gDNA from 14 clinical peripheral blood samples and a blank control in the *JAK2* V617F/Cas12a lateral flow strip assay. Philadelphia-negative myeloproliferative neoplasm (MPN), the patient with essential thrombocythemia; N01–N13, healthy donors; BC, blank control. (**b**) The allele-specific PCR (AS-PCR) results of the gDNA from 14 clinical peripheral blood samples and a blank control. M, GL 2000 DNA maker; MPN, the patient with essential thrombocythemia; N01–N13, healthy donors; BC, blank control.

To evaluate the feasibility of the *JAK2* V617F/Cas12a lateral flow strip assay on the diagnosis of clinical samples, we carried out the analysis of the gDNA extracted from the peripheral blood of 13 healthy donors and one patient with essential thrombocythemia (ET), where the patient was diagnosed with ET according to the 2016 WHO criteria and was demonstrated to have the *JAK2* V617F mutation through next-generation sequencing (NGS). The resulting images are shown in Figure 5a. A clear test line was detected in positive samples; in contrast, no detectable test line was observed from the negative samples. In parallel, as a reference method for *JAK2* V617F detection, AS-PCR was also performed with gDNA extracted from those samples (Figure 5b). The results were consistent with the results of the *JAK2* V617F/Cas12a lateral flow strip assay.

## 4. Discussion

MPNs are hematological malignancies, which seriously threaten human health and place great mental and economic pressure on society and patients. The *JAK2* V617F mutation is one of the key somatic driver mutations associated with MPNs; thus, it is a potential therapeutic target for MPNs [4,5,6,47]. In recent years, the United States Food and Drug Administration (FDA) approved JAK2 inhibitors, including ruxolitinib and fedratinib, for the treatment of MPNs [48,49,50,51,52,53]. Thus, the detection of *JAK2* V617F mutation is of significant social value and practical importance for the diagnosis and treatment of MPNs and allogeneic bone marrow transplantation (allo-BMT).

The sensitivity was reported to reach at least 1–3% for the detection of *JAK2* V617F allele burden, because this threshold was shown to be correlated with pathogenicity and carried important clinical interest [23,54,55]. However, in existing methods, some of these are not sensitive enough to detect a minority of patients with low-level *JAK2* V617F mutation, while some are sufficiently sensitive but yield frequent false-positive signals [23,25]. In the method developed in this study, the maximum attainable sensitivity was 0.01% and the minimum detectable concentration of gDNA could reach 0.01 ng/μL, which is two orders of magnitude higher than the sensitivity required for clinical application. In addition, when it was used to validate clinical samples, there were no false-positive results. Hence, the assay is expected to provide clinicians with a powerful and effective means for companion diagnostic of MPNs.

In the present study, we report for the first time a CRISPR/Cas12a-based nucleic-acid detection system used to detect the *JAK2* V617F mutation. The whole process can be divided into three steps: (1) DNA pre-amplification (PCR or RPA); (2) CRISPR/Cas12a-based target detection (the *JAK2* V617F/Cas12a fluorescence detection system or the *JAK2* V617F/Cas12a lateral flow strip assay); (3) reading and analysis of the results (fluorescence signal or lateral flow strip). Compared with current detection methods for the *JAK2* V617F mutation, our methods offer several features and advantages. Firstly, the sensitivity reached the extremely high value of 0.01%, such that the detection ratio of *JAK2* V617F mutation would not tend to differ between peripheral blood and granulocytes as sample sources [23,56,57]. As a result, the assay did not require isolating granulocytes from peripheral blood. Secondly, in addition to the *JAK2* V617F mutation, there may be other somatic mutations in MPNs patients, such as exon 12 of *JAK2, CALR*, and *MPL* [8,58,59,60,61,62]. Likewise, it is also possible that these different mutations can be assayed by simply adjusting the crRNA, thus embodying the method’s simplicity and versatility. Thirdly, the test process was highly efficient, requiring only 1.5 h. Fourthly, our methods are more economical [34,63]. Fifthly, the *JAK2* V617F/Cas12a fluorescence detection system not only is capable of highly sensitive qualitative detection, but also has the potential of quantitative detection. Lastly, the *JAK2* V617F/Cas12a lateral flow strip assay does not rely on sophisticated instruments and skilled technicians, and it is expected to be further integrated into a miniature portable diagnostic device, enabling its application for on-site, point-of-care, low-resource settings, and even home detection. Accordingly, our assays have obvious advantages in the detection of *JAK2* V617F mutation.

## 5. Conclusions

In this study, we first developed and validated an efficient *JAK2* V617F/Cas12a fluorescence detection system and a *JAK2* V617F/Cas12a lateral flow strip assay for the rapid, specific, sensitive, robust, simple, and economical detection of the *JAK2* V617F mutation, which has significant implications for the diagnosis, treatment, and prevention of MPNs. As a proof of concept, these two systems are adaptable and scalable for detecting somatic mutations in tumor-related genes.

## Supplementary Materials

The following are available online at https://www.mdpi.com/article/10.3390/bios11080247/s1, Figure S1. The sequences of inserts in the recombinant plasmids; Figure S2. Schematic representation of the locations of RPA primers; Table S1. Detailed sequences of primers, crRNAs, and probes in this study.
