# Supplementary material for: CRISPR/Cas12a-Based Ultrasensitive and Rapid Detection of JAK2 V617F Somatic Mutation in Myeloproliferative Neoplasms
Source: Biosensors (Basel). 2021 Jul 24;11(8):247. doi: 10.3390/bios11080247 (PMC8394843; doi:10.3390/bios11080247)
Supplement: Supplementary file 1 [file biosensors-11-00247-s001.zip › Supplementary Materials.pdf]

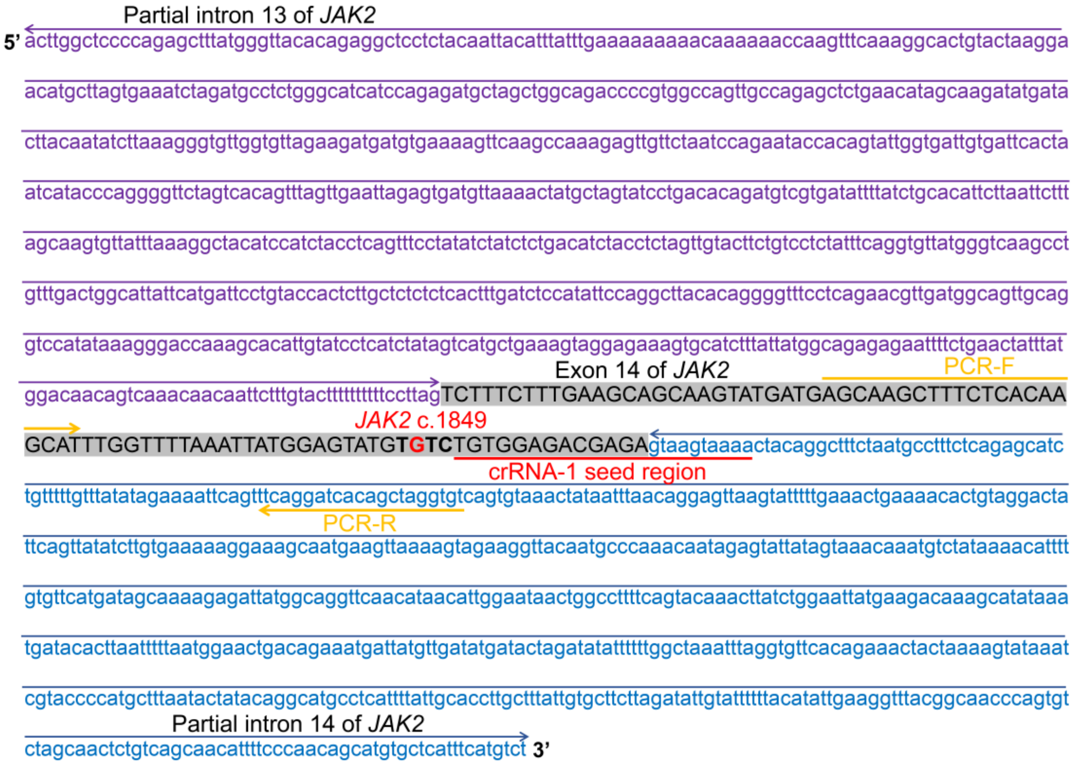

(a)

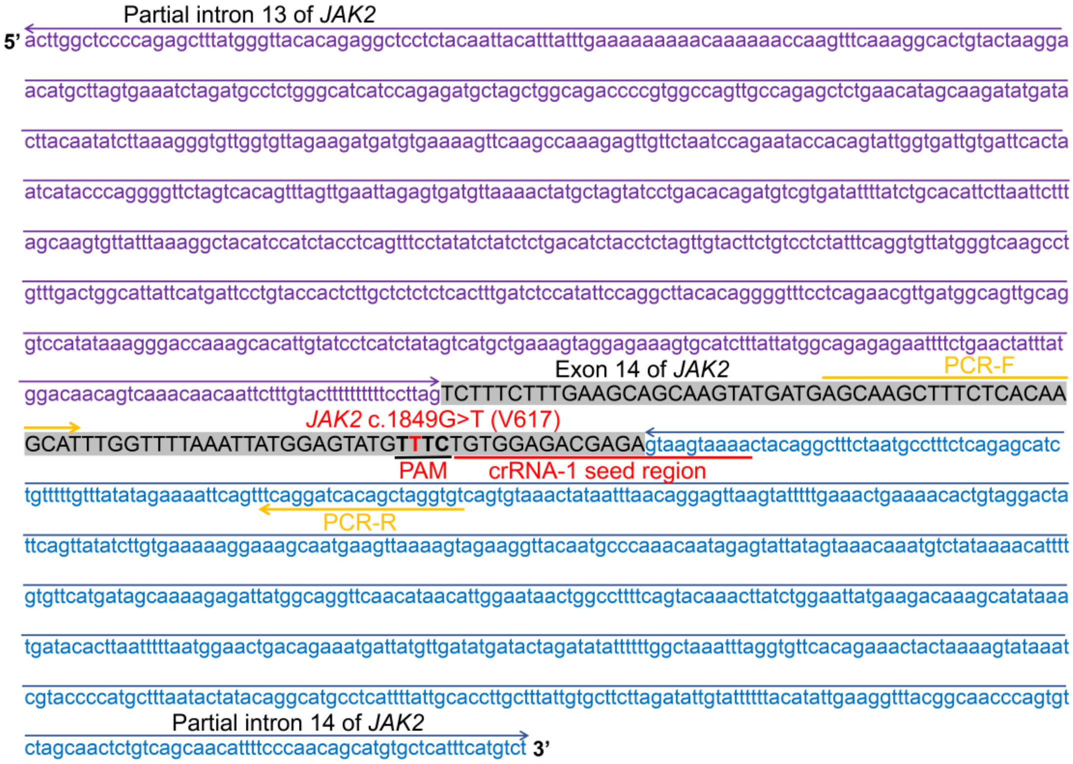

(b)

Figure S1. The sequences of inserts in the recombinant plasmids. (a) The inserted sequence of the wild-type plasmid. The *JAK2* c.1849 was indicated in bold and red. (b) The inserted sequence of the mutant-type plasmid. The *JAK2* c.1849G>T (V617F) was indicated in bold and red. The PAM was in bold and underlined by a black line. The crRNA-1 seed region was indicated with a red line. The exon 14 of *JAK2* was in gray shadow. The partial intron 13 of

JAK2 was in purple and underlined by a purple arrow. The partial intron 14 of *JAK2* was in blue and underlined by a blue arrow. The PCR primers (PCR-F, PCR-R) were indicated by orange arrows.

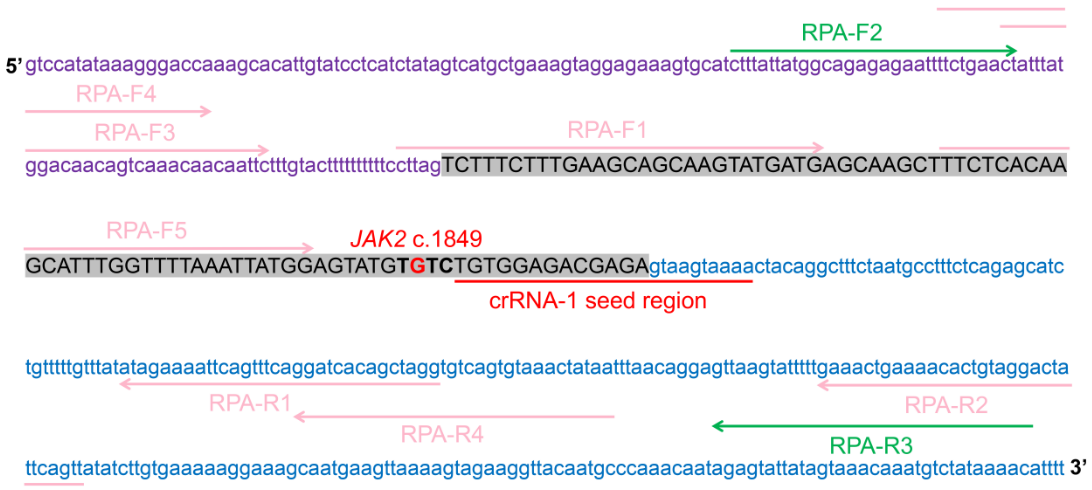

Figure S2. Schematic representation of the locations of RPA primers. The primers RPA-F2 and RPA-R3 used for RPA in this study were indicated by green arrows. The other RPA primers were indicated by pink arrows.

Table S1. Detailed sequences of primers, crRNAs, and probes in this study.

Name	Sequence
**F**	5′-ACTTGGCTCCCCAGAGCTTTATGGGT-3′
**Fm**	5′-TGGTTTTAAATTATGGAGTATGTTTCTGTGGAGACGAG-3′
**R**	5′-AGACATGAAATGAGCACATGCTGTTGGGA-3′
**Rm**	5′-ACTTACTCTCGTCTCCACAGAAACATACTCCATAATTTA-3′
**PCR-F**	5′-AGCAAGCTTTCTCACAAGCAT-3′
**PCR-R**	5′-ACACCTAGCTGTGATCCTGAA-3′
**RPA-F1**	5′-CTTAGTCTTTCTTTGAAGCAGCAAGTATGATG-3′
**RPA-F2**	5′-CTTTATTATGGCAGAGAGAATTTTCTGAACT-3′
**RPA-F3**	5′-CTATTTATGGACAACAGTCAAACAACAATTC-3′
**RPA-F4**	5′-TTCTGAACTATTTATGGACAACAGTCAAACAA-3′
**RPA-F5**	5′-TTCTCACAAGCATTTGGTTTTAAATTATGG-3′
**RPA-R1**	5′-CCTAGCTGTGATCCTGAAACTGAATTTTCTAT-3′
**RPA-R2**	5′-AACTGAATAGTCCTACAGTGTTTTCAGTTTC-3′
**RPA-R3**	5′-CCTACAGTGTTTTCAGTTTCAAAAATACTTAAC-3′
**RPA-R4**	5′-TTATAGTTTACACTGACACCTAGCTGTGATCC-3′
**AS-F**	5′-ATCTATAGTCATGCTGAAAGTAGGAGAAAG-3′
**AS-Fm**	5′-AGCATTTGGTTTTAAATTATGGAGTATATT-3′
**AS-R**	5′-CTGAATAGTCCTACAGTGTTTTCAGTTTCA-3′
**crRNA-1**	5′-UAAUUUCUACUAAGUGUAGAUUGUGGAGACGAGAGUAAGUAAAA-3′
**crRNA-2**	5′-UAAUUUCUACUAAGUGUAGAUAAUUAUGGAGUAUGUUUCUGUGG-3′
**crRNA-3**	5′-UAAUUUCUACUAAGUGUAGAUUGGAGUAUGUUUCUGUGGAGACG-3′
**fluorophore-quencher (FQ) probe**	5′-FAM/TTTTTTTTTTTT/MGB-3′
**FITC-ssDNA-Biotin probe**	5′-FITC/ACACACACACACACACACAC/Biotin-3′
